# Liquid Silicone-Induced Extensive and Debilitating Granulomatosis Responding to Hydroxychloroquine

**DOI:** 10.1155/2019/8173790

**Published:** 2019-07-24

**Authors:** Nargis Jilani, Javeria Shabbir, Elena Nemytova

**Affiliations:** ^1^Medical Resident PGY2 Department of Internal Medicine, Lincoln Medical Center, Bronx, NY, USA; ^2^Medical Resident PGY3 Department of Internal Medicine, Lincoln Medical Center, Bronx, NY, USA; ^3^Board Certified in Internal Medicine and Geriatric, Primary Care Physician, Department of Medicine, Lincoln Medical Center, Bronx, NY, USA

## Abstract

In the last two decades, there has been a significant rise in body-image improvement among the American consumers. Cosmetic injectable procedures have increased by 40.6% in the past 5 years. There has also been an increase in nonmedical, illegal, and self-appointed personnel, offering cheaper hazardous procedures. Silicone has been in use since 1965. In 1991, FDA issued guidelines prohibiting the marketing of injectable liquid silicone. However, it is biologically inert, is associated with inflammatory response, and leads to serious complications like granulomatosis, migration, acute pneumonitis, pulmonary embolism, and even death. Here, we present a case of silicone-induced granulomatosis with extensive migration which ended in bilateral mastectomy, multiple anterior chest debulking procedures, and finally peg tube placement due to compression of the esophageal lumen by granulomas. The patient was eventually started on immunomodulatory treatment, hydroxychloroquine with good response.

## 1. Introduction

According to 2017 survey by American Society of Esthetic Plastic Surgery (ASAPS), there is a 5.1% increase and a massive 40.6% increase in injectable procedures in the past 5 years. These procedures are most common between the ages of 20 and 65. Breast augmentation being the most common surgical procedure, it costs around $3513 to $4014 [1]; this attracted into the field of plastic and esthetic medicine a substantial number of illegal and incompetent operators [2]. Liquid silicone was initially considered inert, but it has been associated with a variety of adverse inflammatory effects [3]. Serious complications have been reported 3 weeks to 23 years after injections. Silicon granuloma is one of the feared complications of liquid silicone use [4]. We present a case of extensive and debilitating granulomatosis after injection of liquid silicon by unprofessional. After years of surgeries, chronic pain management, and courses of corticosteroids, she eventually responded well to hydroxychloroquine treatment.

## 2. Case Presentation

**Figure 1:**
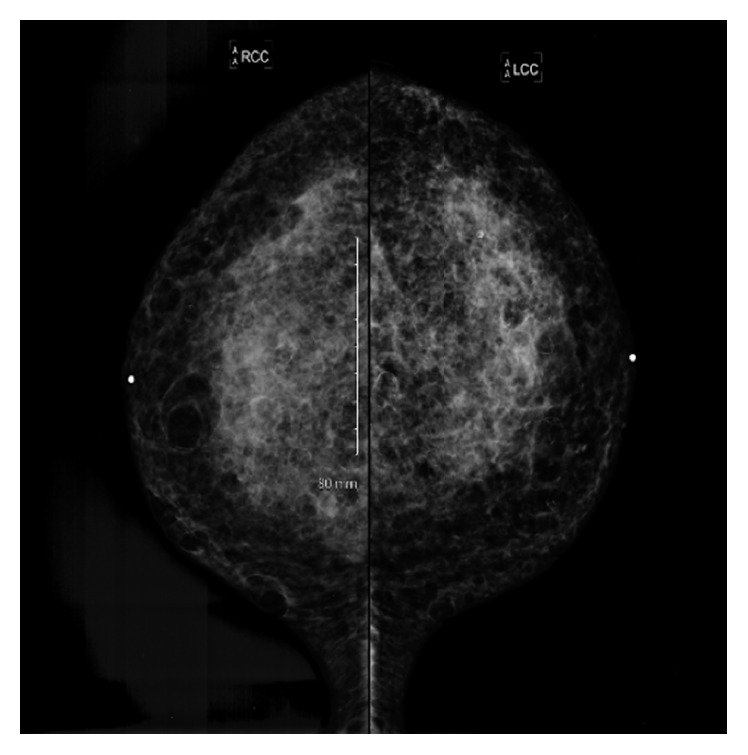
Mammography 2004: scattered benign-appearing calcifications and cysts with rim calcified are present in both breasts.

**Figure 2:**
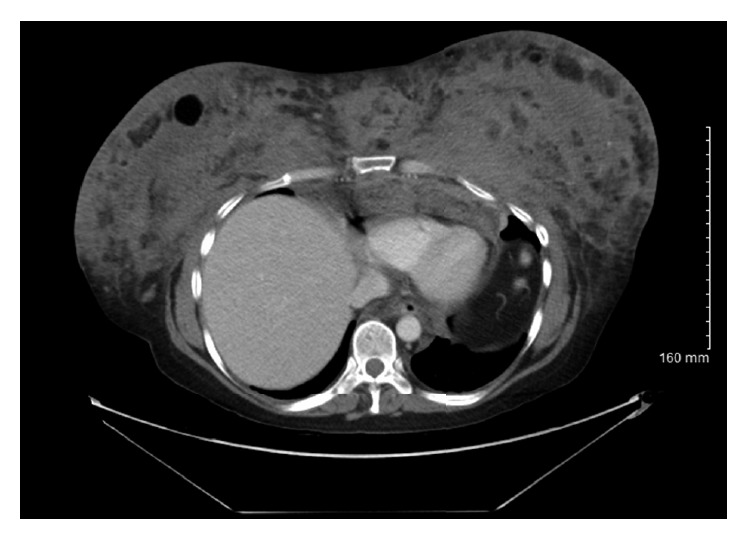
CT chest 2007: breast predominantly fibrous in composition with multiple well-circumscribed areas of fat throughout the breast parenchyma.

**Figure 3:**
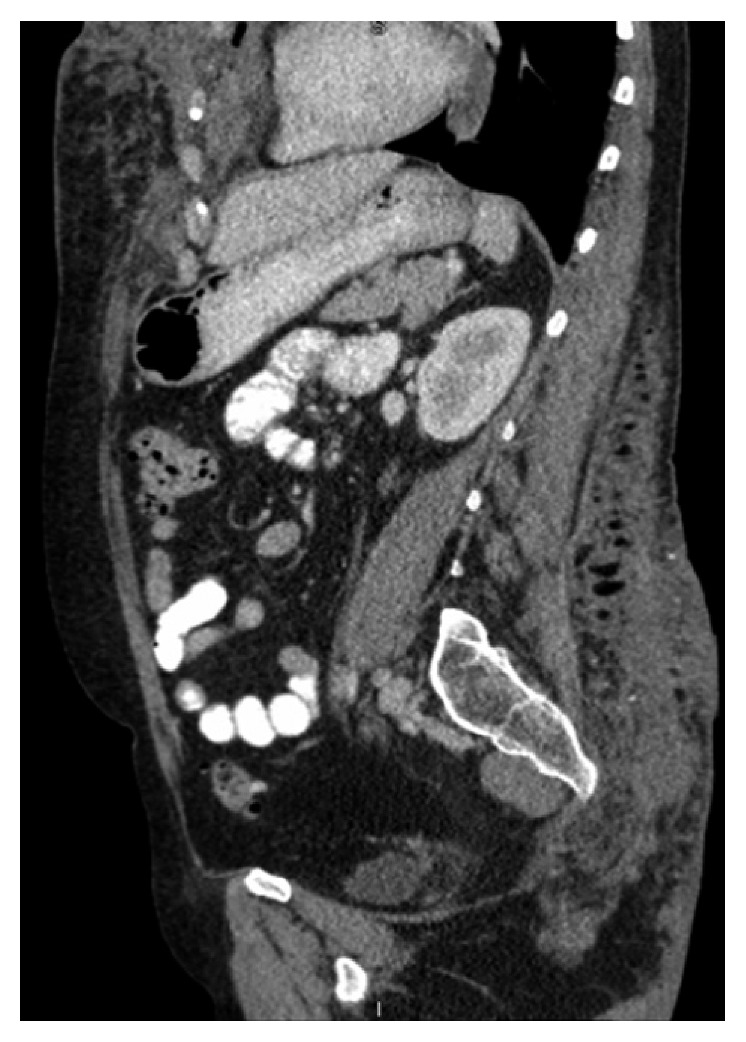
CT abdomen/pelvis 2007: extensive subcutaneous fibrosis involving buttocks.

Our patient is a fifty-one-year-old woman who was initially admitted to our facility in July 2007 with complaints of malaise, intermittent subjective fevers, and bilateral breast pain. She admitted undergoing soft tissue augmentation of her breast and buttocks with unknown “silicone oil” in 2001 by an untrained practitioner in Mexico; mammography showed calcifications in both breasts from 2004 (Figure 1). Physical examination revealed multiple subcutaneous tender “stony consistency” masses with erythema involving her breast, buttocks, thigh, and back. Areas of thickened skin were also noticed in multiple areas involving sternum and thighs. Laboratory workup revealed moderate leukocytosis with left shift. Abdominal and pelvic computed tomography (CT) revealed extensive infiltration of the soft tissue planes involving breast, back, and gluteal areas (Figures 2 and 3). She was treated with ibuprofen 400 mg every 8 hours and a short course of corticosteroids prednisone 40 mg daily with tapering dose for 4 weeks without significant improvement.

**Figure 4:**
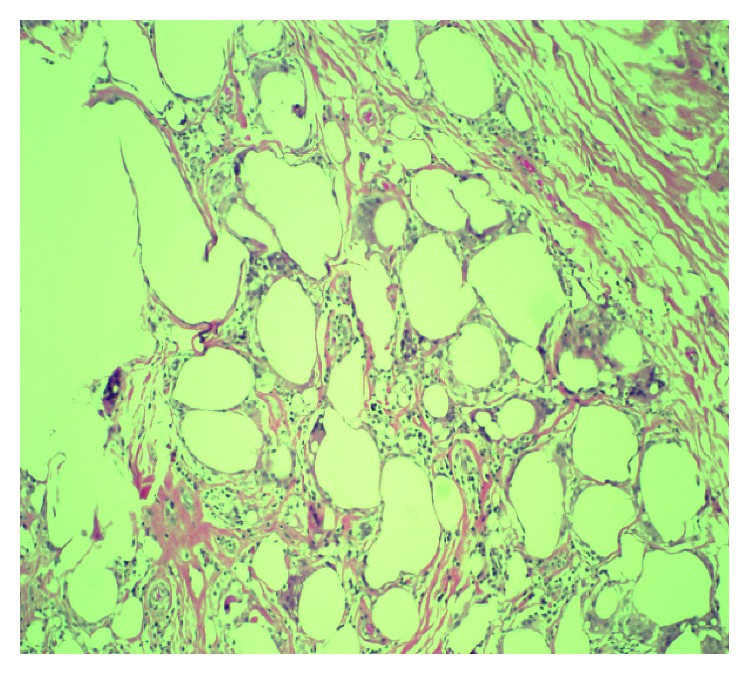
100x magnifications showing fibro fatty tissue with chronic granulomatous inflammation, with lymphocytes, few neutrophils, and multinucleated giant cells.

**Figure 5:**
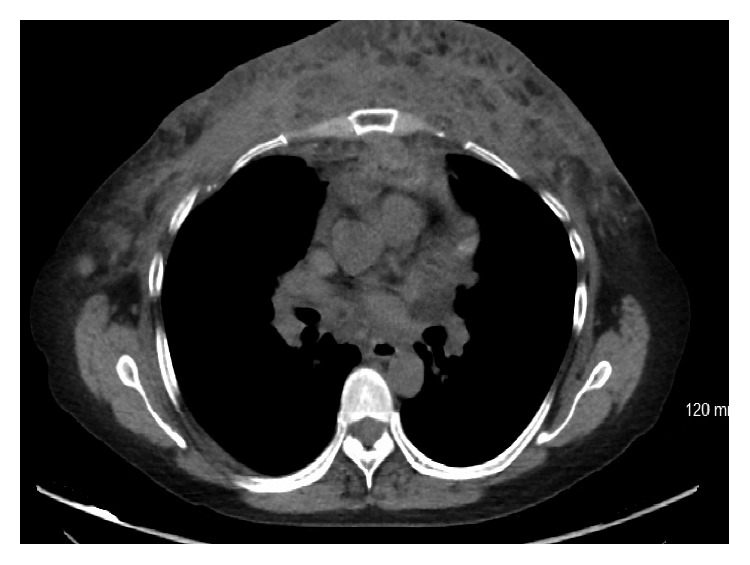
CT chest 2012: extensive Infiltration involving the mediastinum as far back as the spine, most pronounced anteriorly and extending throughout the chest to the diaphragm along with epicardial infiltration.

**Figure 6:**
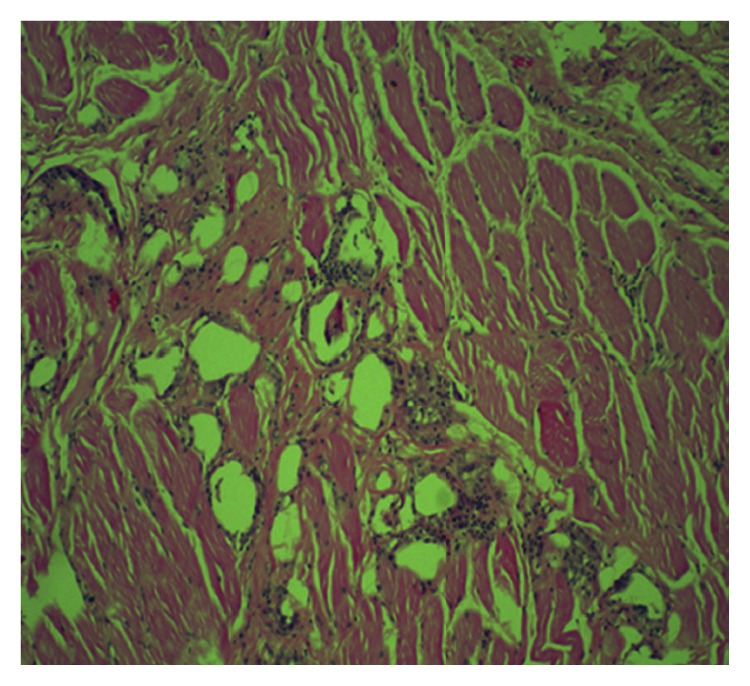
100x magnifications showing skeletal muscle with chronic granulomatous inflammation.

For the next few years, she had multiple admissions due to chronic pain involving breasts, buttock, and back. In 2009, she underwent bilateral mastectomy. The histopathological examination of biopsy specimen of breast showed foreign material reaction with microscopic and macroscopic fibrous loculation, giant cell reaction, and fibrosis (Figure 4). However, she continued to have silicone migration to her neck and anterior chest leading to multiple debulking surgeries in 2011 and 2012, and CT chest still showed extensive infiltrative even after multiple debulking procedures (Figure 5). The histopathological examination of biopsy specimen from anterior chest revealed muscle tissue with fibrosis, fat necrosis, giant cell reaction with chronic inflammation, and calcifications as shown in Figure 6.

**Figure 7:**
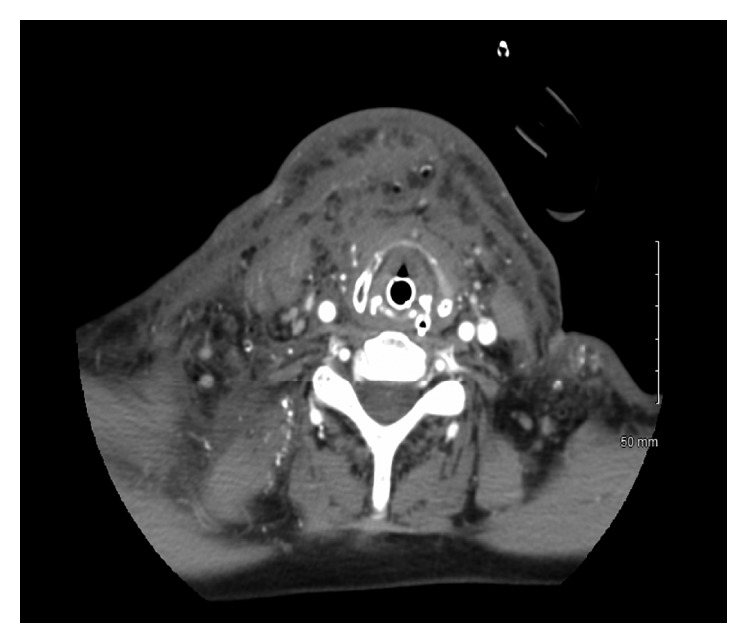
Numerous scattered calcifications and fatty lesions throughout the soft tissues with increased edema in the submandibular and anterior cervical spaces.

She continued to have multiple admissions for pain in her chest and back and swelling in her neck. She was intubated electively in 2015 for airway protection due to increased swelling. CT neck revealed increased edema in submandibular and anterior cervical spaces with increased soft tissue swelling along the musculature. There were numerous scattered calcifications and fatty lesions throughout soft tissue (Figure 7).

**Figure 8:**
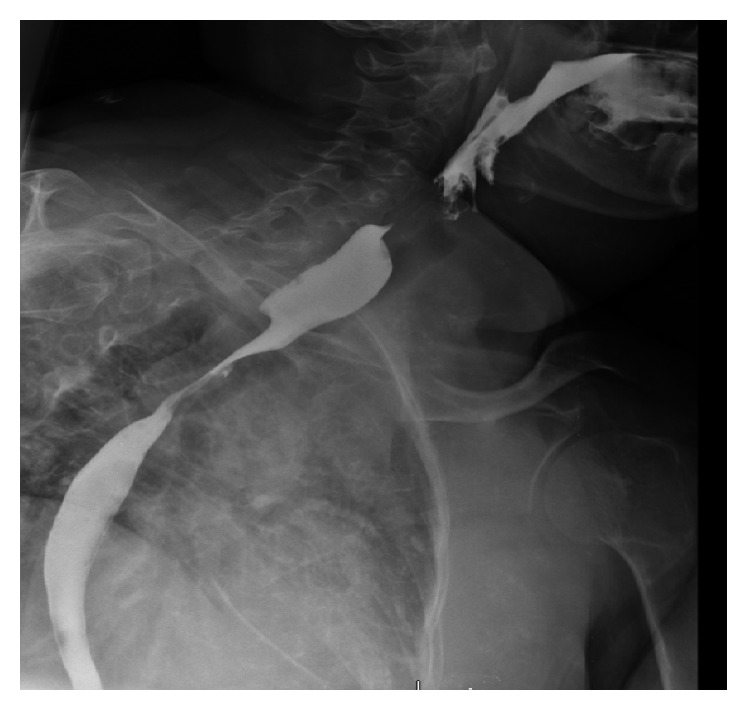
Esophagram 2017: 5 mm curvilinear extraluminal contrast collection adjacent to the proximal esophagus at the level of the clavicles.

She eventually started to develop progressive dysphagia and was admitted to hospital in 2017 for not able to swallow solid food. Esophagram shown in Figure 8 and esophagogastroduodenoscopy revealed a food bolus impaction at 20 cm with severe narrowing of the esophagus due to external compression by granulomatous mediastinitis. She underwent percutaneous endoscopic gastrostomy tube placement.

Patient was evaluated by rheumatology services for the treatment of chronic granulomatous inflammation in October 2015. Rheumatologic workup was negative for rheumatoid factor (RF), antinuclear antibodies (ANA), and anti-dsDNA antibodies, anti-SSA and anti-SSB. Given the signs of extensive systemic inflammation, she was started on oral hydroxychloroquine (HCQ) 200 mg twice daily dose with progressive improvement in her symptoms of pain in next few months and she was kept on HCQ 200 mg twice a day with significant decrease in number of emergency room visits and hospital admissions since 2016 after initiation of hydroxychloroquine. The imaging remained stable with no further progression of granulomatous inflammation to other organs. Also there was a significant improvement in inflammatory markers given below in Table 1.

## 3. Discussion

Liquid silicone (dimethylpolysiloxane) has been in use for the last 5 decades for soft tissue augmentation. It is inert, permanent, thermally stable, and noncarcinogenic and does not support bacterial growth. It is approved by Food and Drug Administration (FDA) for intraocular injection for severe retinal detachment and is used as off label by experienced practitioners as filler for treatment of acne scars, facial rhytides, and severe facial deformities [4, 5]. There is an increase in use of illegal liquid silicone for augmentations of breast, buttocks, hips, and lips in nonmedical facilities by nonprofessionals [5, 6].

The massive volumes of impure and adulterated silicone by illegal unqualified persons can lead to numerous complications, ranging from minor like erythema, edema, and soft tissue infections to serious complications like siliconomas, migration to distant organs causing lymphadenopathy, granulomatous hepatitis, acute pneumonitis, fatal pulmonary embolism, adult respiratory distress syndrome, and even death [2, 7].

Siliconomas initially present as recurrent cellulitis like reactions with pain, induration, nodules, and ulcerations along with localized lymphadenopathy [4]. As in our patient, the diagnosis was made on the basis of clinical history, imaging, and histopathological findings. Histopathology shows multinucleated giant cells and histiocytes containing foamy, empty-looking cytoplasm of variable size representing silicone particles [8]. It can be differentiated from sarcoidosis by observation of crystalline particles of varying sizes mainly in giant cells, using light microscopy, and doubly refractile particles using polarized microscopy. [9].

Granuloma formation is a natural host response to wall off exogenous substance large enough to be ingested by macrophages [6]. The exact pathogenesis of granuloma formation is unknown, and it is believed to be due to T-cell activation leading to high levels of TNF-alpha, which is a proinflammatory cytokine. It plays a role in granuloma formation. The TNF inhibitor etanercept has been used successfully in patient with siliconomas [4].

Hydroxychloroquine (HCQ) has been in use for chronic granulomatous disease like cutaneous sarcoid granuloma and inflammatory disorders. It has numerous immunomodulatory effects. Studies have shown that it causes inhibition of inflammatory cytokines like IL-1-alpha, IL-2, and TNF-alpha. HCQ was started as initial therapy on our patient to decrease inflammatory mediators, as it is a well-tolerated medication in use for over 70 years. [10]. Patient's symptoms improved remarkably within few months of treatment, as evident by markedly decrease in the number of emergency room visits and hospital admissions since 2016. As of now, this is the first case of silicone-induced extensive granulomatosis treated with HCQ with clinical improvement.

## 4. Conclusion

Our patient is a unique presentation of disseminated extensive siliconomas involving anterior chest, neck, back, buttocks, mediastinum, and obstructing esophagus resulting in chronic pain and leading to multiple surgical procedures (bilateral mastectomy, anterior chest debulking surgeries, and PEG tube placement). She finally responded to hydroxychloroquine with a noticeable improvement in her quality of life with 80% improvement of her chronic pain and less repeated admissions to hospital. This helps physicians to start HCQ as initial therapy due to its safety profile.

## Figures and Tables

**Table 1:** 

Erythrocyte sedimentation rate	
September 2009	80
January 2018	25

C-reactive protein	
September 2009	160
July 2018	7.8
